# What are the factors associated with catastrophic health expenditure in Colombia? A multi-level analysis

**DOI:** 10.1371/journal.pone.0288973

**Published:** 2023-07-27

**Authors:** Juan Luis Ramirez-Agudelo, Monica Pinilla-Roncancio

**Affiliations:** School of Medicine, Universidad de los Andes, Bogotá, Colombia; Jimma University, ETHIOPIA

## Abstract

**Introduction:**

Target 3.8 of the Sustainable Development Goals calls for the guaranteeing of universal health service coverage without generating financial risks for households and individuals. In Colombia, there is no up-to-date information on the proportion of households that suffer catastrophic health expenditure (CHE), nor about how these expenses are associated with the place of residence. To contribute to an understanding of these issues, this study analyses the differences in the levels of CHE among Colombian households, and their association with the province and area (urban or rural) of residence.

**Methods:**

This is a descriptive and analytical cross-sectional study using the 2016–2017 National Household Budget Survey, the household and population Census 2018, and the Register of Health Providers 2017. We used the definition of CHE proposed by the World Health Organization, with a threshold of 20%. We estimated the percentage of households facing CHE, and its intensity, and estimated a multi-level logistic regression model, using as the dependent variable the question of whether a household experienced CHE, and the province as a second level, where explanatory variables related to the province were included.

**Results:**

We found differences in CHE levels according to the province of residence. At the national level, 1.77% of households experienced CHE, and households in the provinces of Boyacá (5.04%), Nariño (4.04%), Cauca (3.82%), and Chocó (3.78%) faced the highest CHE. For most households with CHE in these provinces, spending on medicines and medical consultations represented close to 50% of their out-of-pocket spending. The multi-level logistic regression model indicated that there are significant variations in CHE attributed to the provinces under study, where the contextual variables of hospital-bed density (AOR = 0.91; 95% CI 0.86–0.96) and incidence of multi-dimensional poverty (AOR = 1.13; 95% CI 1.01–1.30) were factors associated with CHE. For an urban household, 6.58% of the CHE variation is attributed to the province in question, while for a rural household the corresponding variation is 1.56%.

**Conclusions:**

The geographical location of the household is a key factor when studying CHE in Colombia, where rural households present higher levels of CHE, mainly in the delivery of medicines and medical consultations. The findings reveal the need to analyse financial protection at the local level and establish policies to protect households, especially poor households, from CHE.

## Introduction

Financial protection is crucial for the functioning of any health system, and it constitutes an important aspect of achieving universal health coverage, as presented in target 3.8 of the Sustainable Development Goals (SDGs) [1]. All individuals, irrespective of their social and economic status, should have access to the necessary health services without facing financial risks or compromising their standard of living [1, 2]. Out-of-pocket (OOP) payments, which refer to direct expenses for health care paid by households, are a primary indicator of inadequate financial protection in the health-care sector [3]. OOP payments are considered to be the least efficient and inequitable way of financing health care, as they can prevent households from seeking necessary care and can lead to Catastrophic Health Expenditure (CHE) for those who do seek care [4].

CHE occurs when OOP payments for health-care services exceed a certain threshold of household consumption, indicating that the household may struggle to maintain essential subsistence needs, such as food, due to the financial burden of health-care expenses [5, 6]. CHE is caused not only by high-cost health events or hospitalisation; it can also result from relatively small but constant health-care expenses, such as the purchase of medicines [2]. The number of people worldwide whose CHE exceeded 10% of their household budget rose from 940 million in 2015 to 996 million in 2017. Similarly, the number of people whose CHE exceeded 25% of their household budget increased from 270 million to 290 million during the same period [7]. Studies have found that households in poorer and more unequal countries are more likely to face CHE, as even a small expenditure on health care can pose a high risk of causing a significant financial burden [4, 8, 9]. Additionally, households located in rural areas and those with adults older than 65 and/or young children, as well as those lacking health insurance and having low levels of education, are at the highest risk of facing CHE [10, 11].

The passing of Law 100 in 1993, which created the General System of Social Security in Health (GSSSH), has significantly increased the coverage of, and access to, health services in Colombia [12]. This system includes the contributory and subsidised regimes which are governed by the essential principles of equity, quality, and increased coverage [13]. But despite its efforts, the GSSSH has not been able to guarantee financial protection for all households in Colombia. As a result, inequitable access to health goods and services persists, particularly among those with greater economic and social vulnerability [14, 15]. In Colombia, access, opportunity, and continuity of health services are inequitable and follow a social gradient, with barriers to health-care services increasing as socio-economic status decreases [15]. The inequities in poverty levels, numbers of doctors and nurses, hospital infrastructure, and health-care service availability in different regions of the country are factors that contribute to the gap in quality and opportunity of access to health-care services [16–18].

The most recent data in Colombia analysing the proportion of households facing CHE relate to 2011, when it was estimated that for 9.6% of households their expenditure on health care exceeded 20% of their household budget [19]. According to the study, the incidence of CHE was higher in the Pacific and Atlantic regions of Colombia, with rates of 16.9% and 11.3% respectively [19]. Studies conducted in Colombia that have analysed OOP payments patterns and differences based on geographical, socioeconomic, and demographic characteristics have confirmed that a household’s area of residence is a factor in the incidence of CHE [19–24].

Studies conducted in India, Bangladesh, China, and Malawi have employed multi-level regression models to capture the impact of both household and contextual variables associated with the place of residence on the incidence of CHE [25–28]. In Colombia and other countries of Latin America and the Caribbean, studies have focused on explanatory models at the household level that assess the likelihood of facing CHE [11, 19, 29, 30]. Evidence that quantifies the effect of the geographical location (province, area of residence) on the CHE created is still scarce in Colombia. The use of the multi-level approach in CHE analyses in Colombia would contribute to the study of differences by province, and the interaction with the area (urban or rural) in the occurrence and intensity of CHE.

Research using multi-level analysis would enable an expansion of the evidence in the analysis of social determinants and inequities associated with CHE in Colombia, and thus enable an assessment of the gaps that exist between different places of residence. This study aimed to analyse the association between province and area of residence in the phenomenon of CHE in Colombia, using a multi-level logistic regression model.

## Methods

### Data source

This study is a secondary cross-sectional analysis of the National Household Budget Survey (NHBS) that was conducted between July 2016 and July 2017 by the Colombian National Administrative Department of Statistics (DANE) [31]. Additionally, three sources of information were used to obtain information at the province level: the Integrated Social Protection Information System (SISPRO), the Colombian Population and Household Census, and the Special Registry of Health Service Providers (REPS).

The NHBS was designed by DANE to select households by means of multi-stage stratified cluster sampling. The survey collected information from 87,201 households in various regions and areas of the country. However, the NHBS design only collected information from the urban population in nine provinces of Colombia: Amazonas, Arauca, Casanare, Guainía, Guaviare, Putumayo, Vaupés, Vichada, and San Andrés [31]. Households in these provinces were excluded from our analysis, since the purpose is to analyse the differences between urban and rural contexts within a province of residence; therefore, the final dataset includes a total of 74,330 households in 24 provinces in Colombia.

Information about the incidence of multi-dimensional poverty for each province was taken from the national Census 2018 [32]. SISPRO and REPS were used to obtain the estimated number of health professionals (doctors and nursing staff), the number of hospital beds, and the number of health-service providers in each province of the country in the year 2017 [18, 33]. The data obtained from these sources were merged with the NHBS database, using the province variable as a key.

This study was approved by the Ethics Committee of the Universidad de los Andes, Colombia, in December 2020. We processed the data openly, in accordance with the requirements stipulated by DANE and SISPRO.

### Variables of interest

Out-of-pocket payments (OOP) were defined as the expenses incurred directly by individuals or households when receiving health services [3], and they were calculated as the total monthly expenditure on goods and services related to health. All expenditures reported in the NHBS provided the frequency of purchase (daily, weekly, monthly, quarterly, or annual), and the expenditures were converted to monthly values using the general methodology implemented by DANE [31].

This analysis excluded expenses for dental services and non-monetary expenses. As a robustness analysis, dental services were included in an additional model, which aimed to analyse if OPP payments associated with those increased or not the risk of CHE. The results were consistent with the main results presented in the article, where variables such as the age of the household head, household size, presence of members aged 60 years or older, and the area of residency were significant factors of CHE (S1 Table).

CHE occurs when OOP payments exceed a certain threshold as a proportion of the income or expenditures of a household in a defined period [34]. This study calculated the CHE in accordance with the methodology proposed by the World Health Organization (WHO) [34], with a threshold of 20% of the capacity to pay that has been widely used in other studies in Colombia [19, 21, 23, 24]. Other values (10%, 25%, and 40%) were used as a sensitivity analysis.

The household capacity to pay (CP) was calculated as:

$$CP=\left\{ \begin{array}{l} \exp-se\;if\;se\leq Food\;expenses\\ \exp-Food\;expenses\;if\;se>Food\;expenses \end{array}\right.$$

where *exp* indicates total household spending; *se* indicates household subsistence level; and *Food expenses* is the monthly total food expenditure (excluding alcoholic beverages and food consumed outside the house). Per capita subsistence spending was estimated as the weighted average of food spending per person of households in the 45th and 55th percentiles in terms of health spending; *se* is the product of per capita subsistence spending and the equivalence scale (size of the household raised to 0.56) [9].

A dichotomous variable was constructed based on the fraction of OOP payments divided by CP (OOP/CP), in which CHE took the value 1 if the fraction was greater than or equal to 0.20, and 0 otherwise. The intensity of the CHE is defined as the difference between the proportion of OOP payments relative to the ability to pay and the defined threshold (Z = 0.20, or 0.10, 0.25, and 0.40 for the sensitivity analysis). If the household incurs CHE, then *E*_*i*_ = 1, otherwise *E*_*i*_ = 0:

$$CHE\;intensity=E_{i}\left((\frac{OOP}{CP})-Z\right)$$

The dependent variable of the study is the occurrence of CHE in each household as a dichotomous variable. Categorical variables of the household head were included as explanatory variables: age (<45, 45–59, ≥ 60), sex (female, male), occupation (employed, looking for job, disabled/unable to work, and engaged in other activities), educational level (none, primary, secondary, middle, higher/university), health-insurance type (contributory, subsidised, special, no insurance). For the household characteristics variables, the following categorical variables were included: per capita income quintiles, household size (1–2, 3–4, ≥5), presence of members other than the head of household aged 60 years or older (Yes/No), presence of members aged 5 years or younger (Yes/No), and area of residence (urban, rural). The contextual variables at the provincial level included incidence of multidimensional poverty (MP), number of doctors and nurses, density of hospital beds, and density of health-service providers.

### Statistical analysis

To analyse the factors of the CHE in Colombian households and the effect of the province and area of residence on this expense, a multi-level logistic regression model was employed. A two-level hierarchical model was proposed, with the first level comprising 74,330 households and the second level comprising the 24 provinces of residence that the survey represents in both rural and urban areas. First, we checked whether there was a multi-level structure in the data by estimating the logistic model without any explanatory variable (empty model), leaving the random effect at the province level. Having found that the empty model was significant at the 95% confidence level (random effects account for a significant amount of variation in the CHE between provinces), a bivariate analysis was conducted to assess the association between each explanatory and response variable.

We also checked for multi-collinearity by examining the correlation matrix of the explanatory variables, which showed low to moderate correlations. The normality of random effects was checked by means of a density plot, which showed approximately normal distributions. Finally, we checked for independence by plotting the residuals against the predictor variables and found no evidence of patterns or trends. After verifying the assumptions, the multi-level logistic regression model used in this study to estimate the probability (*π*_*ij*_) of a household facing CHE is given by:

$$\ln\left(\frac{\pi_{ij}}{1-\pi_{ij}}\right)=\beta_{0}+{\gamma X}_{ij}+\delta Y_{ij}+\alpha Z_{j}+\mu_{0j}+\mu_{ij}Urban$$

In this equation, *X*_*ij*_ is a vector containing the characteristics of the head of the household, along with each coefficient (*γ*). Similarly, *Y*_*ij*_ is a vector with the characteristics of the household and their corresponding coefficients (*δ*). *Z*_*j*_ represents the contextual variables at the province level.

The variable *Urban* is a categorical variable that takes the value of 1 if the household is urban and 0 if it is rural. The term *μ*_0*j*_ is the residual at the province level, which reflects the random part of the model. Finally, the interaction term *μ*_*ij*_ *Urban* captures the effect that the area (urban or rural) and province have on each household.

The variance partition coefficient (VPC) was used to measure the variability in the occurrence of CHE at the province level. Given that the interaction between area and province was included in the model, the VPC was calculated separately for urban and rural households, using the following formula:

$$VPC\left\{ \begin{array}{c} Urban:\;\frac{\sigma_{uo}^{2}+2cov(u_{o},{Urban}_{ij})+\sigma_{uij}^{2}}{{(\sigma }_{uo}^{2}+2cov(u_{o},{Urban}_{ij})+\sigma_{uij}^{2})+3.29}\\ Rural:\;\frac{\sigma_{uo}^{2}}{\sigma_{uo}^{2}+3.29} \end{array}\right.$$

where $\sigma_{uo}^{2}$ is the variance associated with the intercept of the model, *cov*(*u*_*o*_, *Urban*_*ij*_), the covariance between the department and the area, and $\sigma_{uij}^{2}$ is the variance of the area. The value of 3.29 in the formula is a constant of the estimated error of a multi-level logistic model calculated as *pi*^2^/3, being the variance of the standard logistic distribution. This type of VPC calculation is an approximation for binary response models; although the VPC is more recommended for continuous variables, the adjustment made for level 2 residual of 3.29 allows this VPC to be used in the study.

All analyses were conducted using the statistical software R Studio version 4.0.4 [35]. Adjusted Odds Ratios (AOR) were calculated for all household and province variables included in the model, and the confidence intervals and significance were assessed at the 95% confidence level. In the multi-level logistic model, minimal differences have been observed in estimates and standard errors from weighted and unweighted multi-level regression [35–37]. Therefore, survey weights were not applied in the calculation of the AOR in the model.

## Results

Table 1 reports the characteristics of household and the head of household in the 74,330 households selected for this study, as well as the statistics of the contextual variables of 24 provinces of the country. It is observed that close to 75.23% of the heads of households are under 60 years of age, and 63.23% of households have a male head. 2.62% of household heads are permanently unable to work due to some physical, mental, or social condition, and 21.34% are not active in the labour market because their main economic activity is studying or doing household chores. Regarding the contextual variables, 12 of the 24 provinces have an incidence of multi-dimensional poverty higher than 8.9%. and half of the provinces have fewer than 29 health professionals (doctors and nursing staff) per 10,000 population.

**Table 1:** Characteristics of the households and provinces in the study.

Level 1: Characteristics of the head of household (Head of the HH)	(n = 74330)	n †	% ‡
*Age*	< 45 years	30275	43.83%
	45–59 years	24002	31.40%
	≥ 60 years	20053	24.78%
*Sex*	Male	43514	63.23%
	Female	30816	36.77%
*Occupation*	Employed	51404	73.29%
	Looking for a job	2399	2.75%
	Disabled, unable to work	2232	2.62%
	Other (outside the labour market)	18295	21.34%
*Level of education*	None	3614	6.18%
	Primary	21076	32.54%
	Half	20923	26.20%
	Secondary	10359	13.97%
	Higher / University	18358	21.12%
*Health insurance*	Contributory	37815	49.10%
	Subsidised	29848	42.41%
	Special	2794	2.71%
	No health insurance	3873	5.78%
**Level 1: Household characteristics**	**(n = 74330)**		
*Size of the household*	1–2 members	24849	33.82%
	3–4 members	33553	44.99%
	≥ 5 members	15928	21.18%
*Number of members ≥ 60 years*	No	62219	85.67%
	Yes	12111	14.33%
*Number of members ≤ 5 years*	No	56024	73.26%
	Yes	18306	26.74%
*Area of residence*	Rural	5754	21.61%
	Urban	68576	78.39%
**Level 2: Province**	**(n = 24)**	Median	
Density of health professionals * 10,*000 inhabitants*		29.0	
Density hospital beds * 10,*000 inhabitants*.		19.7	
Density of health-care providers * 10,*000 inhabitants*		2.9	
*Incidence of multidimensional poverty*		8.9	

**†** Count without survey weights

**‡** Percentage with survey weights

The national prevalence of CHE was 1.77% (1.58%– 1.95%), with a mean intensity of 16.49% (14.08%– 18.90%) which indicates that on average Colombian households with CHE spend 36.49% (20% threshold of the CHE + 16.49% of the intensity) of their payment capacity on OOP payments. The provinces of Nariño (4.04%), Cauca (3.82%), and Chocó (3.78%) present the highest levels of CHE, and these results are significant. When we analysed the density of health facilities and health providers in the country, the results showed that in 50% of the provinces the median of the density of health professionals is less than 29 per 10,000 inhabitants, an aspect that is related to the unequal distribution of health professionals and facilities in the country. In the case of the incidence of multidimensional poverty, the median is 8.9%; therefore 50% of the provinces have levels of multidimensional poverty lower than this number, and others (especially those that are more remote and have a larger percentage of rural areas) present higher levels of multidimensional poverty. We used the median, given the unequal distribution of facilities and professionals in the country.

The sensitivity analysis conducted on the extent and intensity of CHE at different threshold levels produced the expected results. Specifically, increasing the threshold level reduced the percentage of households that experienced CHE, while simultaneously increasing the intensity. At the 40% threshold, national results showed that fewer than 1% of the households faced CHE (0.38%– 0.57%) with an intensity of 21.68% (14.96% –28.4%), and Boyacá is still the province with the highest CHE, with 2.23% (1.28%– 3.85%), but in this case it is significantly different from zero. On the other hand, at 10% threshold 5.04% (4.73%– 5.33%) of Colombian households presented CHE with an intensity of 11.73% (10.61%– 12.86%) (Table 2).

**Table 2:** Levels of CHE and its intensity in Colombian households at 20% (reference), 10%, 25%, and 40% threshold.

	Z = 10%		Z = 20%		Z = 25%		Z = 40%	
**Province (n = 24)**	% CHE [95% CI]	% Intensity [95% CI]	% CHE [95% CI]	% Intensity [95% CI]	% CHE [95% CI]	% Intensity [95% CI]	% CHE [95% CI]	% Intensity [95% CI]
**National**	5.03 [4.73–5.33]	11.73 [10.61–12.86]	1.77 [1.58–1.95]	16.49 [14.08–18.9]	1.2 [1.04–1.35]	18.32 [15.12–21.53]	0.48 [0.38–0.57]	21.68 [14.96–28.4]
Antioquia	5.67 [4.86–6.6]	9.41 [7.53–11.28]	1.69 [1.23–2.32]	12.97 [9.92–16.03]	1.25 [0.86–1.83]	11.7 [8.14–15.25]	0.33 [0.16–0.68]	11.79 [3.16–20.42]
Atlántico	1.33 [0.9–1.98]	7.17 [4.83–9.51]	0.41 [0.13–1.23]	4.47 [2.35–6.6]	0.21 [0.07–0.62]	2.22 [-0.41–4.85]	-	-
Bogotá D.C.	4.05 [3.4–4.83]	9.72 [7.29–12.15]	1.15 [0.85–1.56]	15.42 [10.18–20.65]	0.76 [0.52–1.12]	17.22 [11.21–23.24]	0.39 [0.22–0.69]	14.08 [7.77–20.39]
Bolívar	5.51 [4.11–7.35]	17.24 [2.92–31.57]	1.83 [0.99–3.37]	33.07 [-1.55–67.69]	1.22 [0.61–2.44]	43.96 [-1.87–89.79]	0.48 [0.17–1.31]	84.59 [-14.4–183.57]
Boyacá	10.72 [8.47–13.48]	17.11 [12.06–22.15]	5.04 [3.49–7.22]	21.73 [14.15–29.32]	4.15 [2.76–6.2]	20.99 [12.02–29.96]	2.23 [1.28–3.85]	19.01 [5.34–32.67]
Caldas	6.49 [4.63–9.03]	10.55 [6.42–14.69]	2.31 [1.31–4.01]	11.12 [2.07–20.16]	1.13 [0.5–2.52]	15.67 [-1.36–32.7]	0.34 [0.08–1.33]	28.49 [17.1–39.88]
Caquetá	3.91 [2.62–5.82]	16.33 [6.2–26.47]	1.38 [0.71–2.63]	30.47 [6.56–54.38]	0.92 [0.4–2.1]	39.2 [8.89–69.51]	0.89 [0.38–2.08]	25.25 [-6.14–56.63]
Cauca	8.11 [6.09–10.73]	12.9 [8.35–17.45]	3.82 [2.3–6.28]	13.72 [8.87–18.57]	2.58 [1.34–4.94]	14.25 [10.08–18.41]	0.98 [0.35–2.67]	9.33 [4.34–14.33]
Cesar	3.56 [2.44–5.17]	9.14 [5.65–12.64]	0.92 [0.41–2.04]	11.71 [7.1–16.31]	0.86 [0.37–2.01]	7.28 [2.48–12.09]	0.04 [0.01–0.12]	26.24 [-7.45–59.94]
Chocó	6.88 [3.74–12.33]	18.54 [8.87–28.2]	3.81 [1.79–7.9]	21.04 [5.13–36.96]	2.67 [0.92–7.47]	24.29 [11.28–37.31]	1.87 [0.42–7.91]	17.55 [16.19–18.92]
Córdoba	5.92 [4.18–8.32]	12.27 [7.93–16.61]	2.18 [1.26–3.73]	18.44 [9.88–27]	1.72 [0.91–3.21]	17.76 [8.22–27.31]	0.57 [0.24–1.35]	24.8 [8.27–41.33]
Cundinamarca	3.78 [2.62–5.43]	12.37 [5.66–19.08]	1.24 [0.66–2.3]	18.6 [0.72–36.49]	0.76 [0.37–1.57]	23.53 [-2.49–49.56]	0.25 [0.07–0.84]	49.41 [-5.29–104.12]
Huila	4.43 [2.66–7.3]	8.82 [6.32–11.33]	1.71 [0.79–3.65]	7.9 [2.94–12.86]	0.74 [0.23–2.33]	9.2 [0.69–17.71]	0.24 [0.05–1.23]	4.95 [1.34–8.56]
La Guajira	3.54 [2.24–5.54]	3.75 [2.46–5.05]	0.25 [0.12–0.5]	9.56 [6.5–12.62]	0.18 [0.07–0.46]	7.07 [2.59–11.55]	0 [0–0.03]	52.97 [52.97–52.97]
Magdalena	2.36 [1.51–3.66]	8.85 [4.52–13.18]	0.79 [0.34–1.84]	9.55 [3.82–15.29]	0.67 [0.25–1.76]	5.77 [-0.89–12.43]	0.12 [0.02–0.86]	4.66 [4.66–4.66]
Meta	3.55 [2.52–4.97]	7.92 [5.19–10.65]	0.93 [0.51–1.69]	11.92 [5.77–18.08]	0.54 [0.31–0.94]	13.77 [7.98–19.56]	0.15 [0.05–0.41]	16.06 [8.19–23.94]
Nariño	10.27 [8.49–12.38]	13.32 [9.51–17.14]	4.04 [2.99–5.45]	17.66 [9.55–25.78]	2.6 [1.8–3.73]	21.05 [8.85–33.25]	1.1 [0.59–2.03]	25.96 [2.96–48.96]
Norte de Santander	5.74 [3.9–8.37]	14.09 [8.07–20.11]	2.64 [1.5–4.61]	16.01 [5.15–26.87]	1.35 [0.71–2.58]	23.89 [7.08–40.69]	0.36 [0.12–1.1]	53.42 [35.1–71.74]
Quindío	5.42 [3.68–7.9]	8.26 [6.02–10.49]	1.52 [0.77–2.95]	9.1 [2.9–15.31]	0.55 [0.24–1.24]	16.12 [9.81–22.42]	0.34 [0.11–1.06]	8.05 [2.6–13.5]
Risaralda	2.08 [1.51–2.86]	10.88 [7.37–14.4]	0.79 [0.48–1.28]	12.94 [8.14–17.74]	0.61 [0.35–1.04]	11.06 [5.54–16.57]	0.12 [0.03–0.52]	14.77 [9.46–20.08]
Santander	3.56 [2.67–4.72]	12.22 [8.32–16.12]	1.5 [0.95–2.35]	13.95 [7.93–19.97]	0.94 [0.52–1.72]	16.49 [11.06–21.91]	0.36 [0.13–0.99]	13.04 [0.97–25.1]
Sucre	4.26 [3.06–5.89]	9.35 [5.18–13.53]	1.19 [0.55–2.57]	12.74 [4.98–20.49]	1.02 [0.42–2.49]	9.58 [0.45–18.71]	0.29 [0.06–1.44]	10.46 [9.14–11.79]
Tolima	7.62 [6.12–9.46]	13.14 [9.27–17.01]	3.05 [2.06–4.49]	17.14 [9.97–24.31]	1.98 [1.28–3.05]	20.42 [13.81–27.04]	1 [0.53–1.89]	16.91 [10.81–23]
Valle del Cauca	4.7 [3.96–5.57]	11.58 [8.55–14.6]	1.59 [1.18–2.14]	16.88 [11.42–22.35]	1.1 [0.75–1.6]	18.43 [11.92–24.94]	0.45 [0.25–0.83]	21.21 [12.06–30.36]

**Fig 1:**
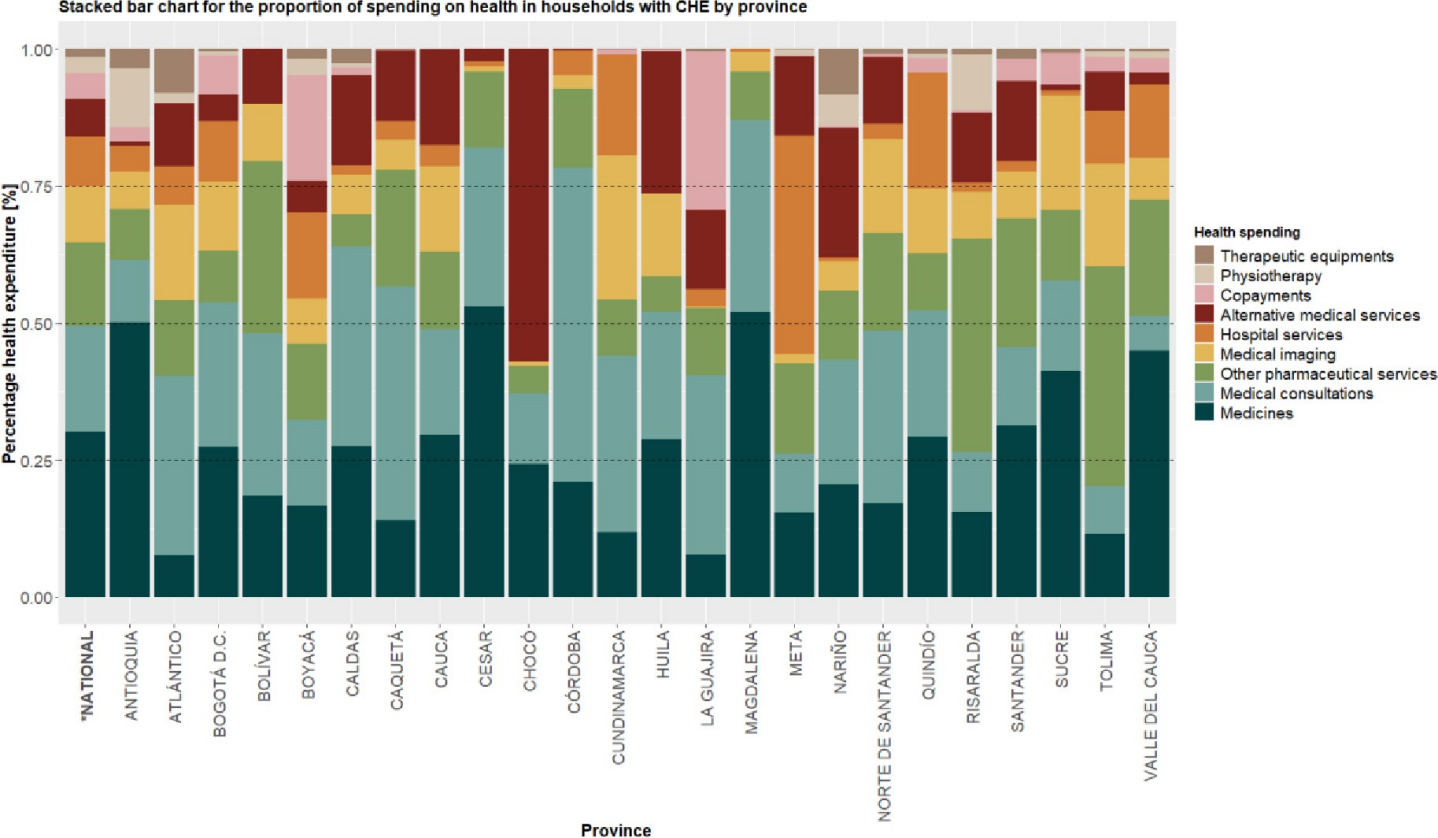
Pattern of out-of-pocket spending on health in households with CHE, by province.

Spending on medicines and medical consultations represents half of OOP payments made by a household with CHE in most of the country provinces (Fig 1). For example, in Antioquia, Cesar, and Magdalena, 50% of OOP payments were allocated to the purchase of medicines. Notably, in Chocó about 60% of OOP payments made by households facing CHE related to alternative medical services, such as homeopathic, bioenergetic, and other non-traditional medicines.

### Factors associated with catastrophic health expenditure

Table 3 presents the results of the multi-level logistic regression model analysing which factors are associated with CHE. The estimated random effect of the province level was statistically significant, indicating that there is a variation in the explanation of the CHE between provinces in Colombia. For rural households, 1.56% of the CHE is explained by the province of residence, and for urban households the comparable percentage is 6.58%. Various household-level factors are associated with the risk of incurring CHE. Indeed, as the age of the head of the household increases, the probability of experiencing CHE also increases significantly. For instance, households with a head aged 60 years or older have a 40% higher chance of experiencing CHE compared with households with a household head younger than 45 years (AOR = 1.40, CI = 1.13–1.73). Households with a female household head have a 34% higher probability of experiencing CHE compared with male-headed households (AOR = 1.34, CI = 1.16–1.54).

**Table 3:** Multi-level logistic regression model for the explanation of CHE in Colombia.

	Adjusted OR [CI 95%]
Constant	0.05 [0.01–0.15] ***
**Level 1: Households (n = 74330)**	
Age of head of household (< 45 years)	
45–59 years	1.26 [1.05–1.51] *
≥ 60 years	1.40 [1.13–1.73] **
Sex of the head of household	
Women	1.34 [1.16 –-1.54] ***
Occupation of head of household (employed)	
Looking for a job	1.75 [1.24–2.47] **
Incapacity to work	2.92 [2.26–3.79] ***
Other work-related activities	1.42 [1.20–1.68] ***
Education level of head of the household (none)	
Secondary	0.69 [0.52 –-0.93] **
Primary	0.91 [0.72–1.16]
High school	0.64 [0.47–0.86] **
Higher education	0.79 [0.58–1.07]
Health insurance of head of household (Contributory)	
Special regime	0.87 [0.60–1.27]
No health insurance	1.27 [0.95–1.70]
Subsidised regime	0.87 [0.73–1.03]
Income quintiles (I)	
II	0.79 [0.65–0.97] *
III	0.65 [0.52 –-0.81] ***
IV	0.65 [0.52–0.82] ***
V	0.77 [0.60–0.99] *
Size of household (1 or 2 members)	
3–4 members	0.62 [0.53–0.72] ***
≥ 5 members	0.45 [0.36–0.57] ***
Household with members aged 60 or older (No)	
Yes	1.61 [1.37–1.90] ***
Household with children 5 years or younger (No)	
Yes	0.89 [0.72–1.10]
Urban areas	0.35 [0.26–0.49] ***
**Level 2: Department (n = 24)**	
Density of health-care professionals	1.02 [0.99–1.04]
Density of hospital beds	0.91 [0.86–0.96] ***
Density of health-care providers	1.25 [0.93–1.67]
Multidimensional poverty incidence	1.13 [1.01–1.30] *
Multidimensional poverty incidence squared	0.99 [0.98–0.99] *
**Random effects**	
$\sigma_{uo}^{2}$	0.052
$\sigma_{uij}^{2}$	0.314
*cov*(*u_o_, Urban o_ij_*)	-0.067
**VPC Rural**	**1.56%**
**VPC Urban**	**6.58%**
Log Likelihood	-4714.4
LR Chi 2	67.5

*P-value < 0.05

** p- value < 0.01

*** p- value < 0.001

Household heads currently identified as employed have a significantly lower risk of exposure to CHE compared with those looking for a job, or with a recognised incapacity to work, or engaged in other occupations. For instance, households where the head is permanently unable to work have a 192% higher probability of exposure to CHE compared with households where the head is employed (AOR = 2.92, CI = 2.26–3.79). Furthermore, households whose head has been educated to high-school level have significantly lower chances of exposure to CHE compared with households with an uneducated head. Hence, households whose head has completed secondary-level education have a 36% lower probability of exposure to CHE compared with households with an uneducated head (AOR = 0.64, CI = 0.47–0.86). As expected, as the quintile of per capita income increases, the probability of experiencing CHE decreases compared with quintile I. Finally, the composition of the household is also associated with CHE; for example, households with five or more members have a 55% lower probability of experiencing CHE compared with households with only one or two members (AOR = 0.45, CI = 0.36–0.57), and households with at least one member aged 60 or older have a 39% higher probability of experiencing CHE compared with households without any members older than 60 years (AOR = 1.61, CI = 1.37–1.90).

The location of the household is a factor associated with the risk of CHE. Specifically, an urban household is 65% less likely to experience CHE compared with a rural household (AOR = 0.35, CI = 0.26–0.49). A one-unit increase in the density of beds in the province reduces the probability of experiencing CHE by 9% (AOR = 0.91, CI = 0.86–0.96). We included two variables capturing the relationship between the incidence of multi-dimensional poverty and CHE. The quadratic relationship of the MP incidence of the province and the CHE indicates that households in provinces where 12% or more of the population are multi-dimensionally poor present a lower probability of incurring CHE than households in provinces with an incidence lower than this threshold. This is because provinces with lower incidences of poverty are associated with higher numbers of available resources, where individuals can seek health services and face CHE. Instead, provinces with higher levels of multidimensional poverty are usually those with lower numbers of health providers and higher levels of deprivations, where households usually do not have the opportunity to demand health services, and therefore are likely to face CHE. This does not mean that living in multi-dimensionally poor provinces is a protective factor in terms of CHE, but that living in poor provinces is a factor that reduces the opportunity to look for health services; furthermore, this is a variable captured in the national multidimensional poverty index.

## Discussion

This article has calculated the percentage of households facing CHE in Colombia and analysed how this percentage varies between provinces. The results reveal that fewer than 2% of households in Colombia faced CHE in 2018. However, there are significant differences in the incidence and intensity of CHE across provinces. Also, households experiencing CHE incur the greatest out-of-pocket expenditures because of their need to purchase medicines and pay for medical consultations. The multi-level model revealed that the province is a significant factor explaining the occurrence of CHE. This highlights the association between factors such as the density of hospital beds, the incidence of multidimensional poverty, and the occurrence of CHE in Colombia.

The levels of CHE in Colombia are a subject of vital importance for the health system, since they are related to the financial protection of households [19, 23]. Although there have been substantial improvements in the coverage of health services, there are still gaps in equitable access to health services in certain regions and areas of the country [14, 15].The findings of this study suggest that the percentage of persons facing CHE is relatively low in Colombia. However, the geographical location is important when analysing CHE. For example, the provinces of Nariño, Cauca, Boyacá, and Choco present the highest incidences of CHE, and this result can be attributed to a high proportion of the population living in rural areas. Other authors have also identified that one of the main factors that determine CHE is the urban or rural location of the household [10, 19, 20, 25]. However, it is important to recognise that people living in rural areas also face higher barriers to accessing health services [12, 14, 15, 21], and this may be associated with a lower probability of facing CHE, given that it is not possible for them to have timely access to health services, and when they do, the diseases or illness may be more advanced or more difficult to manage: an aspect that increases their chances of experiencing CHE. In addition, given the barriers that people in rural areas face, if they seek health care from private providers, the costs are usually paid OOP and increase the risk of CHE.

OOP payment patterns showed not only that CHE is the result of payments for high-cost and complex services, but that a high proportion of CHE is the product of the purchase of medicines and medical consultations. OOP payments for medicines have been documented in previous studies in Colombia [20, 22], and they are an expense that becomes catastrophic due to the frequency of the purchase [38]. According to the Insurance Bulletin of the Ministry of Health and Social Protection in Colombia, in 2010 34.7% of the people who were prescribed medications did not receive them, and by 2016 this proportion was close to 36.6% [12]. The lack of timely and continuous delivery of medicines, mainly due to geographical restrictions, can lead to households having to pay for them directly in order to treat a health-related event.

Certain characteristics of the household are factors that might increase the risk of facing CHE. For example, households whose head is older than average are more likely to demand health services, and the risk of diseases increases, which increases the probability of CHE [10, 19, 23]. This finding is also consistent with the fact that other members of the household aged 60 or older might demand greater access to health services that may or may not be covered by insurance, leading to a greater risk of facing CHE [9, 10, 23, 39]. The presence of a female head of household is also a factor that increases that household’s risk of CHE, because the more inequitable social position of women with respect to that of men results in a lower ability to pay, which leads to the OOP payments in these households eventually becoming catastrophic [10, 21–23]. The risk of CHE is reduced if heads of household are in paid employment, and in households in the highest wealth quintiles and in households with more members, since they have higher income levels, which increase the capacity to pay [9, 10, 23, 39].

Unlike in other studies, the presence of children aged 5 years or younger was not significant in explaining CHE [19, 21, 22]. This finding may be due to the improvement in access to health services in Colombia that is specifically targeted at this population group: initiatives such as the National Program for the Prevention, Management and Control of Acute Respiratory Infection (ARI), which reduced the ARI mortality rate from 36.3% in 1998 to 16.5% in 2010 for every 100,000 children under 5 years of age [39].

Contrary to what has been reported in other studies, the health-insurance regime of the head of the household was not found to be a factor determining exposure to CHE [23]. A possible explanation is due to the fact that in Colombia the problem is no longer coverage of health insurance, or the type of health insurance, but actual access to health services [40]. With the unification of the benefit plan and the regulation of the Statutory Health Law 1751 in 2015, people served by all insurance regimes had the same offer of health services [41]. However, due to geographical difficulties, households located in more isolated places have less access to health services, even if they have health insurance [42].

To the best of our knowledge, no study in Colombia or Latin America has evaluated the relationship between the place of residence and the risk of CHE, using the multi-level methodology. However, different studies in Asia and Africa have found that the place of residence is associated with CHE. For example, a systematic review analysing the factors associated with CHE in Sub-Saharan Africa found that living in rural areas was a factor increasing the risk of CHE [43]. Another systematic review found that 18 studies analysed the area of residence as a potential determinant of CHE, and 12 found a significant association between living in rural areas and CHE [10]. Comparing these results with the research findings for Colombia, where for an urban household the department or province in question explains 6.58% of the variability of the CHE, and for a rural household the corresponding figure is 1.56%, it can be assumed that the level and heterogeneity in a cluster as broad as the department reduces the explanation percentage of the CHE. This is one of the main limitations of the study, where level 2 is very broad and heterogeneous. However, the survey data did not allow an analysis at a lower level. Despite this limitation, the variation found at the department or province level continues to be relevant and significant, quantifying the differences between rural and urban households in the explanation of Colombia’s CHE.

The results at the province level showed that the density of hospital beds per 10,000 inhabitants and the incidence of multi-dimensional poverty are contextual factors that affect exposure to CHE. The increase in the number of beds, such as for surgery, emergencies, and intensive care, reduces the risk that a household in a certain province will face CHE. This can be explained by the fact that better availability of beds improves access to health services, which reduces the risk that a household must make direct payments to access health care [42, 44]. In addition, the relationship between multidimensional poverty in the province and the probability of CHE indicates that, for provinces with very high levels of poverty, the incidence of CHE in the department is lower. This is explained by the fact that low levels of CHE in the province do not necessarily mean that there are high levels of financial protection [45].When a department has an incidence of multidimensional poverty greater than 12%, poor households experience higher deprivations in access to health services, which increases their poverty and also reduces the risk of households facing CHE, given that they did not have the opportunity to access health services. Therefore, the results reveal that in the case of the poorest provinces, households are less likely to present CHE, but this is associated with the lack of access to health care.

Although the results of this study are important and contribute to the discussion on CHE, the study has its own limitations, related to the survey design and data. In the first place, the cross-sectional design does not allow the analysis of CHE over time. Therefore, it must be clear that the CHE may relate to households experiencing fortuitous events in the period in which they were consulted, and that the payments do not necessarily correspond to long-term situations [22, 37]. Secondly, the information provided by individuals, especially in terms of income and expenses, might be affected by problems associated with recall periods, which might overestimate or underestimate the real value. Another important limitation is the lack of information on people’s particular health conditions, such as the presence of chronic non-communicable diseases, or indirect expenses such as travel to a health centre. An additional limitation is the level of analysis of the multi-level model. Even though the province can capture some heterogeneity, given the differences between municipalities that belong to each of the provinces, it is not possible to capture all the variability of the data. The final limitation is that, given the type of study, it is not possible to talk about causal effects of the area of residence and CHE.

It is recommended that for future research information should be collected at the municipal level, and preferably with longitudinal data that allow a better study of CHE patterns and their determinants. Likewise, the analysis of dental expenses requires special attention, given the pattern of payments incurred for such services in the highest quintiles of wealth. An analysis of the impact of oral care on OOP payments would contribute to an analysis of inequity in this type of service and would assess how even households with higher income levels are experiencing very high expenses, which may even become catastrophic, as demonstrated by other studies [46, 47].

## Conclusions

The results of this study identified the differences in CHE experienced in different provinces of Colombia. The existing inequality in CHE levels relates mainly to conditions experienced by urban and rural households, whereby in the latter case the problem does not result from levels of health-service coverage but from problems of access to the necessary services due to geographical restrictions, such as the timely delivery of medicines. Financial protection should not be analysed exclusively for households with a higher incidence of CHE: it is also essential to consider the geographical department in question. These findings are important evidence to support policy decisions in the field of public health, where interventions are made to protect households in the different departments from financial shocks. Reducing the gaps in service provision between urban and rural districts, and addressing the deep inequities in access to health services between departments in Colombia, are ways to reduce catastrophic health expenditures.

## Supporting information

S1 Table Results of the multi-level logistic regression including dental services. (DOCX)
