# Supplementary material for: What are the factors associated with catastrophic health expenditure in Colombia? A multi-level analysis
Source: PLoS One. 2023 Jul 27;18(7):e0288973. doi: 10.1371/journal.pone.0288973 (PMC10374149; doi:10.1371/journal.pone.0288973)
Supplement: S1 Table — (DOCX) [file pone.0288973.s001.docx]

**Table 1. Results of the multi-level logistic regression including dental services**

| **Variable** | **Model 1 †** | **Model 2 ††** | **Model 3 †††** |
| --- | --- | --- | --- |
|  | **Adjusted OR [CI 95%]** | **Adjusted OR [CI 95%]** | **Adjusted OR [CI 95%]** |
| Constant | 0.034 [0.023-0.05]*** | 0.180 [0.078-0.413]*** | 0.181 [0.081-0.405]*** |
| **Level 1: Households (n = 87,057)** | | | |
| Age of head of household ( < 45 years) |  |  |  |
| 45–59 years | 1.347 [1.133-1.602]*** | 1.350 [1.135-1.605]*** | 1.350 [1.135-1.606]*** |
| ≥ 60 years | 1.515 [1.237-1.854]*** | 1.521 [1.243-1.862]*** | 1.519 [1.240-1.860]*** |
| Sex of head household |  |  |  |
| Women | 1.248 [1.094-1.425]** | 1.247 [1.093-1.424]** | 1.248 [1.093-1.425]** |
| Occupation of household head (ref. category working) |  |  |  |
| Searching for job | 1.594 [1.16-2.191]** | 1.581 [1.150-2.172]** | 1.583 [1.151-2.175]** |
| Incapacity to work | 2.968 [2.33-3.780]*** | 2.953 [2.318-3.761]*** | 3.012 [2.363-3.839]*** |
| Other | 1.399 [1.194-1.638]*** | 1.392 [1.189-1.631]*** | 1.400 [1.195-1.640]*** |
| Education level of head of the household |  |  |  |
| Secondary | 0.703 [0.536-0.924]* | 0.700 [0.532-0.918]* | 0.710 [0.540-0.934]* |
| Primary | 0.925 [0.738-1.158] | 0.914 [0.730-1.144] | 0.919 [0.733-1.152] |
| High school | 0.649 [0.485-0.868]** | 0.644 [0.481-0.860]** | 0.649 [0.485-0.869]** |
| Higher education | 0.785 [0.586-1.050] | 0.778 [0.581-1.040] | 0.789 [0.589-1.057] |
| Health insurance of head of household (contributory) |  |  |  |
| Special | 0.902 [0.636-1.281] | 0.905 [0.637-1.285] | 0.897 [0.631-1.273] |
| No health insurance | 1.338 [1.022-1.751]* | 1.347 [1.029-1.764]* | 1.349 [1.031-1.767]* |
| Subsidised | 0.874 [0.741-1.031] | 0.882 [0.748-1.040] | 0.877 [0.743-1.035] |
| Income quintiles |  |  |  |
| II | 0.779 [0.645-0.942]** | 0.776 [0.642-0.937]** | 0.777 [0.643-0.939]** |
| III | 0.666 [0.543-0.818]*** | 0.663 [0.540-0.814]*** | 0.659 [0.537-0.810]*** |
| IV | 0.649 [0.522-0.808]*** | 0.648 [0.521-0.806]*** | 0.645 [0.519-0.803]*** |
| V | 0.753 [0.594-0.956]* | 0.754 [0.594-0.957]* | 0.750 [0.591-0.952]* |
| Size of household (1 or 2 members) |  |  |  |
| 3–4 members | 0.646 [0.559-0.746]*** | 0.646 [0.559-0.746]*** | 0.646 [0.559-0.747]*** |
| ≥ 5 members | 0.449 [0.362-0.558]*** | 0.451 [0.363-0.559]*** | 0.451 [0.364-0.560]*** |
| Household with members aged 60 or older |  |  |  |
| Yes | 1.631 [1.396-1.905]*** | 1.629 [1.394-1.903]*** | 1.631 [1.396-1.906]*** |
| Household with children 5 years or younger |  |  |  |
| Yes | 0.927 [0.762-1.129] | 0.930 [0.764-1.131] | 0.928 [0.762-1.129] |
| Urban areas | 0.362 [0.301-0.434]*** | 0.371 [0.309-0.445]*** | 0.375 [0.277-0.506]*** |
| **Level 2: Department (n = 33)** | | | |
| Density of health-care professionals | - | 0.987 [0.976-0.999]* | 0.988 [0.976-0.999]* |
| Density of hospital beds | - | 0.959 [0.924-0.995]* | 0.957 [0.923-0.991]* |
| Multi-dimensional poverty incidence | - | 0.957 [0.934-0.980]*** | 0.957 [0.934-0.981]*** |
| **Random effects** | | | |
| \| 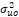 \| \| --- \| | 0.287 | 0.152 | 0.168 |
| \| 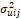 \| \| --- \| | - | - | 0.300 |
| 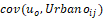 | - | - | -0.136 |
| **VPC Rural** | **8.03%** | **4.43%** | **4.85%** |
| **VPC Urban** |  |  | **5.64%** |
| Log Likelihood | -5345.3 | -5335.5 | -5329.2 |
| LR Chi 2 | 137.22*** | 79.09*** | 91.65*** |
| *****P-value < 0.05, ****** p- value < 0.01, ******* p- value < 0.001 | | | |
| †No contextual variables, †† With contextual variables, ††† With contextual variables and the interaction rural/urban and department | | | |
